# Comparative analysis of drug response and gene profiling of HER2-targeted tyrosine kinase inhibitors

**DOI:** 10.1038/s41416-020-01257-x

**Published:** 2021-01-21

**Authors:** Neil T. Conlon, Jeffrey J. Kooijman, Suzanne J. C. van Gerwen, Winfried R. Mulder, Guido J. R. Zaman, Irmina Diala, Lisa D. Eli, Alshad S. Lalani, John Crown, Denis M. Collins

**Affiliations:** 1 National Institute of Cellular Biotechnology, Dublin City University, Glasnevin, Dublin, Ireland; 2 Netherlands Translational Research Center B.V., Kloosterstraat 9, 5349 AB Oss, The Netherlands; 3 Puma Biotechnology, Inc., 10880 Wilshire Boulevard, Suite 2150, Los Angeles, CA 90024 USA; 4 Department of Medical Oncology, St Vincent’s University Hospital, Dublin, Ireland

**Keywords:** Tumour biomarkers, Targeted therapies

## Abstract

**Background:**

Human epidermal growth factor 2 (HER2/ERBB2) is frequently amplified/mutated in cancer. The tyrosine kinase inhibitors (TKIs) lapatinib, neratinib, and tucatinib are FDA-approved for the treatment of HER2-positive breast cancer. Direct comparisons of the preclinical efficacy of the TKIs have been limited to small-scale studies. Novel biomarkers are required to define beneficial patient populations.

**Methods:**

In this study, the anti-proliferative effects of the three TKIs were directly compared using a 115 cancer cell line panel. Novel TKI response/resistance markers were identified through cross-analysis of drug response profiles with mutation, gene copy number and expression data.

**Results:**

All three TKIs were effective against HER2-amplified breast cancer models; neratinib showing the most potent activity, followed by tucatinib then lapatinib. Neratinib displayed the greatest activity in *HER2*-mutant and *EGFR*-mutant cells. High expression of *HER2*, *VTCN1*, *CDK12*, and *RAC1* correlated with response to all three TKIs. DNA damage repair genes were associated with TKI resistance. *BRCA2* mutations were correlated with neratinib and tucatinib response, and high expression of *ATM*, *BRCA2*, and *BRCA1* were associated with neratinib resistance.

**Conclusions:**

Neratinib was the most effective HER2-targeted TKI against *HER2*-amplified, -mutant, and *EGFR*-mutant cell lines. This analysis revealed novel resistance mechanisms that may be exploited using combinatorial strategies.

## Background

The human epidermal growth factor 2 (*ERBB2*/HER2) gene is frequently amplified or mutated in cancer.^1^ *HER2* gene amplification and/or protein overexpression has been well described in breast and other cancers, and numerous HER2-targeting therapies have been developed and approved. More recently, the advent of next-generation sequencing has facilitated the discovery of a spectrum of somatic *HER2* gene mutations that serve as oncogenic drivers and can be therapeutically targeted.^2,3^ Several HER2-targeted therapies have been developed and approved for the treatment of HER2-positive (HER2+) breast cancer over the past three decades. HER2-targeted therapies can be broadly divided into three categories: the monoclonal antibodies trastuzumab (Herceptin) and pertuzumab (Perjeta), the antibody–drug conjugates trastuzumab emantasine (T-DM1, Kadcyla) and trastuzumab deruxtecan (DS-8201, Enhertu), and small molecule tyrosine kinase inhibitors (TKIs).^4–7^ Trastuzumab plus pertuzumab and chemotherapy are the established first-line therapy for early and advanced HER2+ breast cancer.^8^ Three HER2-targeted TKIs, lapatinib (Tykerb/Tyverb), neratinib (Nerlynx), and tucatinib (Tukysa), have been approved for the treatment of HER2+ breast cancer after progression following HER2-targeted therapy.^7,9,10^ These drugs are all orally available and target the kinase domain of HER2. Lapatinib, the first Food and Drug Administration (FDA)-approved HER2-targeted TKI, is a reversible inhibitor of both epidermal growth factor receptor (EGFR) and HER2 and is approved for the treatment of advanced HER2+ breast cancer following progression on previous therapy, in combination with capecitabine.^9^ Neratinib is an irreversible inhibitor of EGFR, HER2, and HER4 and is FDA approved for the adjuvant treatment of early-stage HER2+ breast cancer after 1 year of trastuzumab treatment.^10^ Neratinib also received FDA approval for the treatment of metastatic HER2+ breast cancer in patients who received two prior lines of HER2-directed therapies.^11^ Tucatinib was recently approved by the FDA for the treatment of patients with metastatic HER2+ breast cancer in combination with trastuzumab and capecitabine.^12^ Tucatinib displays a higher selectivity for HER2 over the other HER family members, in comparison to lapatinib and neratinib.^13^ However, neratinib, an irreversible pan-HER inhibitor, is more potent in biochemical assays. Lapatinib is a reversible inhibitor like tucatinib but has the advantage of targeting both EGFR and HER2, unlike the HER2-specific tucatinib.^6,13^ As the number of therapies available to treat first-line treatment-refractory HER2+ breast cancer grows, it is important to understand the factors that differentiate these clinically approved TKIs.

Currently, the only patient selection and clinical marker of response to these TKIs is HER2+ disease and there is known heterogeneity within HER2+ tumours.^14^ Mutations in *HER2* have also been linked to differential sensitivity to lapatinib and neratinib and resistance to trastuzumab.^15^ This study aims to provide a direct comparison of anti-proliferative activity of the three TKIs clinically approved for HER2+ breast cancer and assess the activity of lapatinib, neratinib, and tucatinib across multiple cancer types in a 115 cell line panel to identify novel potential biomarkers of TKI response outside of HER2 amplification. Novel predictive drug response biomarkers were identified through cross-analysis of drug response with mutation, copy number variation, and gene expression data. These biomarkers could be used to further personalise treatment for HER2+ breast cancer patients or uncover new indications for HER2-targeting TKIs.

## Methods

### Chemical inhibitors

Neratinib was made available by Puma Biotechnology, Inc. Lapatinib (L-4899) was purchased from LC Laboratories. Tucatinib (200291) was acquired from MedKoo. Compounds were stored as dry powders at room temperature in the dark and dissolved in dimethyl sulfoxide (DMSO) before experiments. For dose–response testing, the compound stock was diluted in √10-fold steps in DMSO to obtain a 9-point dilution series, followed by further dilution in aqueous buffer and culture medium.

### Cancer cell lines

A cancer cell line panel of 115 cancer cell lines was utilised in this study (Supplementary Table S1: overview of 115 cell lines and 102 cell lines that were used for comparative profiling and 99 cell lines that were used for gene expression analysis). All cancer cell lines were purchased from the American Type Culture Collection (ATCC) (Manassas, VA), except II-18, which was acquired from RIKEN BioResource Research Center (Tsukuba, Ibaraki, Japan), and HEC-1, HEC-6, and HEC-251, which were purchased from Japanese Collection of Research Bioresources Cell Bank (JCRB) (Ibaraki city, Osaka, Japan). All cell lines were propagated in the cell culture media as recommended by the providers of the cell lines. The experiments were carried out within ten passages of the original vials.

### Cell line authentication and DNA sequence analysis

The authenticity of the ATCC cell lines has been confirmed by short tandem repeat analysis at ATCC and sequence analysis of 25 cancer genes from samples generated at Netherlands Translational Research Center B.V.^16^ In addition, in 24 cell lines, including the RIKEN and JCRB cell lines, the mutation status of the relevant target genes *EGFR*, *HER2*, and *HER3* was determined by Illumina next-generation sequence analysis of genomic DNA isolated from the cell lines at the same passage at which the proliferation assays were performed. To cover all known mutations, as reported in the Broad Institute Cancer Cell Line Encyclopedia (CCLE),^17^ a total of 18 regions in *EGFR*, *HER2*, and *HER3* were selected, and custom oligonucleotides were designed and synthesised at Illumina (Eindhoven, The Netherlands; oligonucleotide sequences detailed in Supplementary Table S2). Genomic DNA was extracted using the AllPrep DNA/RNA Mini Kit (Qiagen, cat. no. 80204), according to the manufacturer’s protocol. DNA was quantified with Quanti-iT Picogreen (Thermo Fischer, cat. no. P11496). Amplicons of the selected regions, with an average length of 138 bp, were prepared by PCR and subsequently partly digested using the Ampliseq Library Plus Kit (Illumina, cat. No. 20019102) according to the manufacturer’s protocol. A sequencing library was prepared after ligation of the amplicons to sample specific bar-coded dual indexing sequencing adaptors (Illumina, cat. no. 20019105). Sequencing was performed on a MiniSeq System (Illumina, San Diego, CA) at 150 bp read length in paired-end mode. Mutation analysis was performed using the FastQC and DNA Amplicon software packages from the BaseSpace Sequence Hub application of Illumina.

### Cell proliferation assays

Sensitivity to neratinib, lapatinib, and tucatinib was examined in cell proliferation assays as previously described.^16^ In brief, cells were seeded in 384-well plates and incubated overnight. Serial dilution of drug was added to the plate and cells were incubated for a further 72 h. Cell proliferation was measured by ATPlite 1Step (PerkinElmer). Luminescence was quantified on an Envision multimode reader (PerkinElmer). Percentage growth was calculated relative to untreated controls. IC_50_ values were calculated by non-linear regression using IDBS XLfit 5. For all analyses, ^10^logIC_50_ values (in nmol/L) were used.

### Comparative clustering analysis of HER2-targeted TKI and other targeted therapy responses

The ^10^logIC_50_ values of neratinib, lapatinib, and tucatinib on 102 cancer cell lines (Supplementary Table S1) were compared to the profiles of 168 anti-cancer agents that have been profiled on the same cell panel.^16^ Correlations were calculated by the Pearson method in R and visualised in a network tree using the Fruchterman–Reingold algorithm, in the package igraph. Two profiles with Pearson correlation (*ρ*) >0.5 are considered similar and are connected with a line in the network tree. Compounds connected to neratinib, lapatinib, or tucatinib were retained. Hierarchical clustering was performed using the Ward method and 1 − *ρ* as measure of distance as described.^18^ Different colours in the network tree represent different clusters identified by the hierarchical clustering.

### Drug sensitivity distribution across disease types

Cell line tissue and disease types were annotated according to the Cellosaurus knowledge resource^19^ and grouped according to the OncoTree classification. The colorectal cell lines were further divided according to a consensus classification based on gene expression.^20^ Drug sensitivity data were visualised in boxplots for disease types represented by at least two cell lines.

### Genetic biomarker analysis

Mutation analysis was carried out in order to determine markers of inhibitor response. Cell lines were classified as genetically ‘altered’ if at least one allele was altered by point mutation, insertion, deletion, or copy number variation, that is, gene amplification or deletion of essential parts of a gene. The mutation status of cell lines was obtained from the Catalogue of Somatic Mutations in Cancer (COSMIC) and CCLE database. In order to ensure that alterations reported by the databases were relevant to cancer growth and drug response, all mutations must be reported as a hotspot mutation in Cancer Hotspots, described as oncogenic, gain of function, or being involved in drug response in OncoKB or Jax CKB, or be described as pathogenic in the literature. Mutation status of *EGFR*, *HER2*, and *HER3* were confirmed by targeted sequencing. The relationship between drug sensitivity and genetic alterations was examined by four different methods. First, a type II analysis of variance (ANOVA) was performed in R. The ANOVA analysis covered 40 genetic alterations, including the most commonly altered mutations in oncogenes and tumour-suppressor genes.^18^ Cell lines harbouring either *EGFR* mutations/amplifications or *HER2* mutations/amplifications were analysed as four separate groups. Second, Mann–Whitney *U* test in R was performed to determine correlations between cell line sensitivity and all cancer gene mutations that occur in at least three cell lines. This resulted in 117 different cancer genes, plus *EGFR* and *HER2* mutation/amplification. An adjusted *p* value of <0.2 was considered significant. Third, the effect on sensitivity of the presence of 20 clinically actionable genes with validated hotspot mutations or copy number variations was examined by type II ANOVA analysis. All *p* values were subjected to Benjamini–Hochberg multiple testing correction. The first three types of analysis are unbiased. The fourth method specifically analysed *HER*-mutated cell lines by Mann–Whitney *U* test to determine whether HER alterations alone are markers of response to each HER2-targeted TKIs.

### Gene expression analysis

Gene expression profiles of 99 of the 115 cell lines (Supplementary Table S1) for 18,900 genes were obtained from CCLE. Pearson correlations were calculated between ^10^logIC_50_ values and gene expression, with associated *p* values determined. In order to understand whether a significant correlation is specific to the particular inhibitor, a Sigma (Σ) score was calculated based on the correlations for each gene with 168 reference compounds that were previously screened in the same cell line panel.^18^ Correlations were validated with the CCLE RNAseq,^21^ COSMIC CLP (v86), and the Genentech^22^ mRNA expression databases, which contain data for 104, 105, and 79 cell lines, respectively.

### Gene Set Enrichment Analysis (GSEA)

The ranked list of 18,900 correlations was used as input for pre-ranked GSEA using the GSEA 4.0.3 software.^23^ Settings were set to default, and the MSigDB 7.1 CGP set was selected as gene set collection.

## Results

### DNA sequence analysis of target gene mutations in the cancer cell line panel

The anti-proliferative effects of neratinib, lapatinib, and tucatinib were examined across a panel of 115 cancer cell lines (Supplementary Table S1). The panel contained 22 cell lines harbouring point mutations or amplifications of the *HER2* (*n* = 9), *HER3* (*n* = 10), or *EGFR* (*n* = 10) genes (Table 1), which encode known targets of one or more of the studied TKIs.^18,24^ Six of the 22 cell lines had multiple HER family alterations. Because genetic variations among different cultures from the same cell line is common and further genetic drift can occur upon culturing, the presence of mutations in *HER2*, *HER3*, and *EGFR* was verified by targeted deep DNA sequence analysis. Sequencing results were compared to the CCLE, which contains sequence data of all cell lines, except II-18 and DLD-1. The mutation status of II-18 was compared to literature,^25^ whereas DLD-1 is known to share the same genetic background with HCT-15.^26^ Our DNA sequence analysis confirmed the presence of an L858R EGFR mutation in this cell line (Supplementary Table S3). Consistent with the genetic overlap of DLD-1 and HCT-15, the same three-point mutations in *HER3* were identified in both DLD-1 and HCT-15 and at almost the same allele frequencies (Supplementary Table S3). DNA sequencing results for all other cell lines also showed very good correspondence to the CCLE data, including highly similar frequencies of the mutant alleles in the cell lines. This confirms the use of the same original source of the cell lines at NTRC and the Broad Institute (i.e., ATCC or JCRB) and that genetic variation was minimal. The only exception was an I655V mutation in HER2, which was identified in six cell lines and is not reported in CCLE. The most likely explanation for this discrepancy is that CCLE performs filtering to remove germline variants.^27^ The functional consequence of the I655V mutation has been the subject of intense debate. Whereas no effect of the mutation on cell proliferation or viability was demonstrated in vitro,^28^ a recent meta-analysis identified a correlation between the HER2 I665V mutation and susceptibility to breast cancer.^29^

**Table 1:** HER2-targeted TKI response in HER-altered cancer cell lines. Comparison of the IC_50_ values for neratinib, lapatinib, and tucatinib in the 22 *EGFR*-mutant, *EGFR*-amplified, *HER2*-mutant, *HER2*-mutant, and *HER3*-mutant cancer cell lines.

### Neratinib is a more potent inhibitor of proliferation across cancer types than lapatinib or tucatinib

**Fig. 1:**
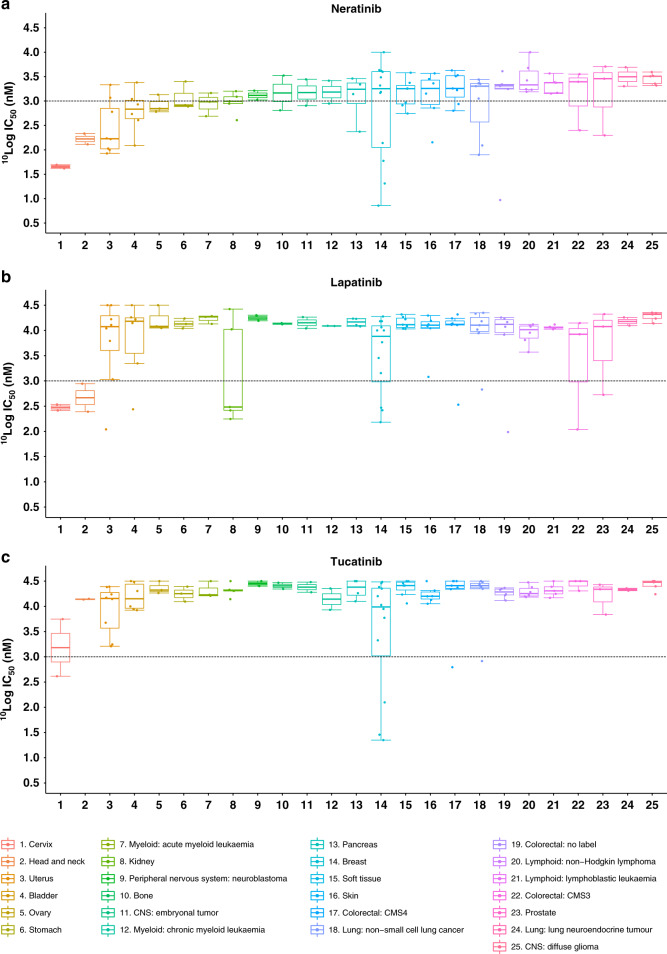
Cellular profiling of HER2 TKIs. Profiling of Neratinib (**a**), Lapatinib (**b**), and Tucatinib (**c**) across a panel of 115 cancer cell lines encompassing 25 different cancer types. Cancer types are ranked from most to least sensitive to Neratinib by ^10^logIC_50_ values. Black dotted line indicates 1 µM.

The cell panel used to profile neratinib, lapatinib, and tucatinib incorporated cell lines derived from 25 different tumour tissues (Fig. 1a–c). Previous studies have highlighted the higher potency of neratinib compared to lapatinib.^30^ Based on IC_50_ values, neratinib displayed the most potent anti-proliferative effect of the three HER2-targeted TKIs, displaying on average 7 and 14 times higher potency than lapatinib and tucatinib, respectively. Neratinib showed the most cancer-type agnostic activity, with 19 of the 25 cancer tissue types included in the study having at least one cell line achieve a sub-micromolar IC_50_ value. In contrast, 11 of the 25 cancer types had nanomolar response to lapatinib and 4 out of 25 for tucatinib.

**Fig. 2:**
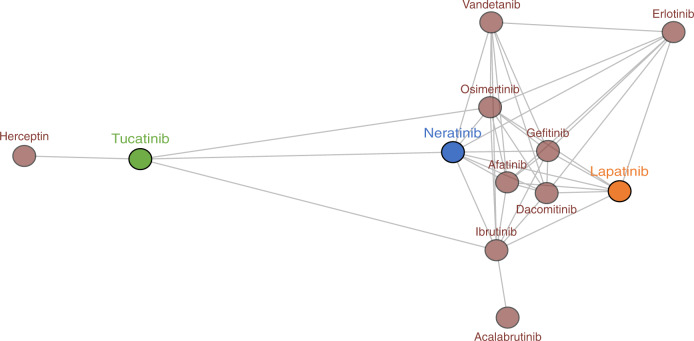
IC_50_ value fingerprint profiling of HER2 TKIs. Comparison of the cellular inhibition profile of 12 kinase inhibitors, including EGFR and HER2 inhibitors. Neratinib (blue), Lapatinib (orange), and Tucatinib (green) were tested on 115 cancer cell lines. The other inhibitors were profiled on 102 cancer cell lines. ^10^logIC_50_ profiles were compared by Pearson correlation. Compounds (circles) were connected when the Pearson correlation was >0.5.

To determine the overall similarities and differences of the three HER2 TKIs and other anti-cancer agents, we compared their IC_50_ fingerprints in the cancer cell line proliferation assays with those of 168 reference agents, including cytotoxic chemotherapies and many targeted inhibitors with diverse mechanisms of actions.^16^ The IC_50_ profiles of the three HER2 TKIs showed significant similarity with other TKIs and not with cytotoxic drugs, confirming the selective nature of the therapies (Fig. 2). Neratinib and lapatinib clustered with several EGFR and dual EGFR/HER2 inhibitors. The profile of tucatinib was most similar to the anti-HER2 antibody trastuzumab, consistent with the selectivity of tucatinib for HER2 in biochemical kinase assays.^13^

**Fig. 3:**
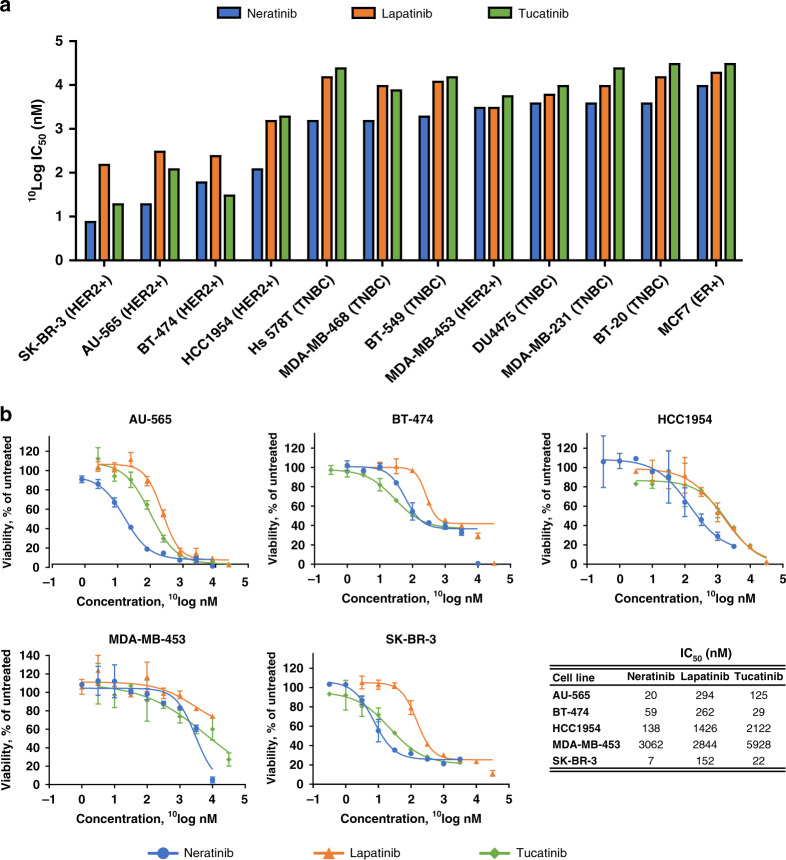
Response of breast cancer cell lines. **a** ^10^LogIC_50_ values of Neratinib (blue), Lapatinib (orange), and Tucatinib (green) in the panel of breast cancer cell lines. Cell lines are ranked from most to least sensitive to Neratinib. **b** Dose–response curves of Neratinib (blue), Lapatinib (orange), and Tucatinib (green) in HER2-positive breast cancer cell lines and corresponding IC_50_ values.

Cross-comparison of TKIs in breast cancer cell lines, given that all three TKIs are approved for clinical use in the treatment of HER2+ breast cancer, the anti-proliferative inhibitory potency of the TKIs on the 12 breast cancer cell lines from the 115-cell line panel was compared. The cell lines included HER2+, ER+, and triple-negative breast cancer subtypes (Fig. 3). As expected, all three TKIs showed preferential activity against HER2+ cell lines. The MDA-MB-453 cell line was the only HER2+ cell line that had a poor response to each TKI. Neratinib was more potent than lapatinib in all but one (MDA-MB-453) of the cell lines and more potent than tucatinib in 11 of the 12 breast cell lines, with BT474 being the exception.

### Cell lines with genetic alterations in HER family genes are more sensitive to neratinib than wild-type cells

Cell lines with alterations in the *HER* genes, i.e., having amplification of or mutations in *EGFR*, *HER2*, or *HER3* genes, were significantly more sensitive to neratinib compared to *HER* wild-type cell lines (*p* < 0.0001; Table 1 and Supplementary Fig. S1). Lapatinib and tucatinib were also significantly more potent in cell lines with genetic alterations in the *HER* genes compared to wild type (*p* = 0.006 and 0.0002, respectively; Fig. 3b, c). As expected, all *HER2*-amplified cell lines, which do not include the HER2+, yet non-*HER2*-amplified, MDA-MB-453 breast cancer cell line, were highly responsive to the HER2-targeted TKIs (Table 1 and Supplementary Fig. S1). Neratinib was on average the most potent TKI in proliferation assays with HER2-amplified cell lines, with lapatinib 11-fold and tucatinib 4-fold less sensitive. In *HER2*-mutant cell lines, neratinib showed nanomolar potencies, whereas lapatinib and tucatinib were active in the micromolar range. The juxtamembrane HER2^R678Q^ mutant bladder cancer cell line J82 was the only cell line that achieved a neratinib IC_50_ value >1 µM. Interestingly, cell lines harbouring point mutations in *EGFR* were almost 11 times more sensitive to neratinib (geometric mean neratinib IC_50_ values = 93.1 nM, *n* = 6) than *EGFR*-amplified cell lines (geometric mean neratinib IC_50_ value = 981.2 nM, *n* = 3). This includes the lung cancer cell line NCI-H1975 that has an EGFR^T790M^ mutation, which is associated with resistance to first- and second-generation EGFR inhibitors.

Cell lines with mutations in *HER3* varied considerably in sensitivity to neratinib. Of the ten *HER3*-mutant cell lines, five harboured an *EGFR* mutation or *HER2* mutation or amplification as well (Table 1). Two of the three HER3^R475W^ cell lines (HEC-1 and HEC-1-B) showed good response to neratinib. However, both cell lines also had *HER2* mutations. Likewise, both cancer cell lines (SKBR3 and AU565) that were HER3^E952Q^ mutant were highly sensitive to neratinib were also *HER2* amplified. HER3^M91I^ UM-UC-3 and HER3^D297H^ FaDu cell lines were moderately sensitive to neratinib, whereas the two cell lines (DLD-1 and HCT-15) that have three mutations in *HER3* were relatively resistant to all three HER2-targeted TKIs. Therefore, the role of *HER3* mutations in neratinib sensitivity remains unclear.

### Analysis of genetic biomarkers of response to HER2-targeting TKIs

**Fig. 4:**
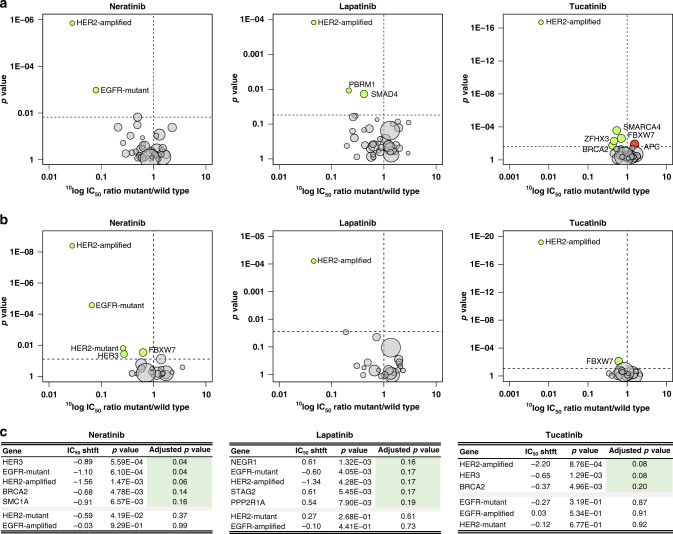
Genomic biomarker analysis. **a** ANOVA analysis of 38 cancer-associated genes in the cancer cell line panel. Genes significantly associated with drug sensitivity are coloured in green (IC_50_ value ratio <1) and those with resistance in red (IC_50_ value ratio >1). An adjusted *p* value < 0.2 was deemed significant. EGFR and HER2 alterations were divided into amplified and mutated to further delineate effect. **b** A shortlisted gene list that were identified as clinically relevant or activating was also examined by ANOVA analysis. **c** Genes of which alterations were significantly correlated with Neratinib, Lapatinib, and Tucatinib response were determined by Mann–Whitney *U* test analysis with Benjamini–Hochberg multiple testing correction analysis of 119 cancer-related genes from COSMIC CLP v80 or CCLE.

In order to identify potential genetic markers of response to the TKIs across the 115 cell line panel, the growth response to each TKI was correlated in an unbiased manner to mutations or copy number variations in 38 well-known oncogenes and tumour-suppressor genes, including *EGFR* and *HER2*, by a type II ANOVA.^16^ *HER2* amplification was identified as a drug response marker for all three TKIs, as expected for HER2-targeted therapies (Fig. 4a). In addition, cell lines with *EGFR* mutations were significantly more sensitive to neratinib than *EGFR* wild-type cell lines. *FBXW7*, *ZFHX3*, *BRCA2*, and *SMARCA4* mutant cell lines were also associated with sensitivity to tucatinib to a lesser degree. *APC*-altered cell lines emerged as less responsive to tucatinib. *PBRM1* and *SMAD4* alterations were both identified as sensitivity markers of lapatinib response. When a reduced list of clinically relevant genes was examined, *HER2* mutations emerged as a significant sensitivity marker for neratinib (Fig. 4b).

Analysis was then performed on a larger set of 117 cancer genes that included genes where genetic alterations occurred in at least three cell lines in the panel to reveal possible novel response markers. The analysis confirmed *HER2* amplification as a drug response marker for all three TKIs (Fig. 4c). Mutation in *EGFR*, *HER3*, *SMC1A*, and *BRCA2* were identified as response markers for neratinib. In this analysis, *EGFR*-mutant cell lines were also more sensitive to lapatinib, whereas *HER3*- and *BRCA2*-mutant cell lines were relatively more sensitive to tucatinib, similar to neratinib. *NEGR1*, *STAG2*, and *PPP2R1A* were identified as resistance markers of lapatinib.

### Correlation analysis of gene expression and HER2-targeting TKIs

The drug response of the three TKIs in the cell line panel was correlated to the expression of 383 clinically actionable genes,^16^ with the aim of identifying new gene expression biomarkers of drug response. To filter out frequently correlated genes, that is, ‘false positives’, Pearson correlations of gene expression and TKI sensitivity were cross-compared to the correlations identified with the 168 anti-cancer agents that were also used for the comparative profiling. Data on basal gene expression levels of 99 of the 115 cell lines in the panel were available at the CCLE and downloaded for the analysis. To validate the identified gene expression-based biomarkers, analyses were repeated with three different independently generated data sets.

**Fig. 5:**
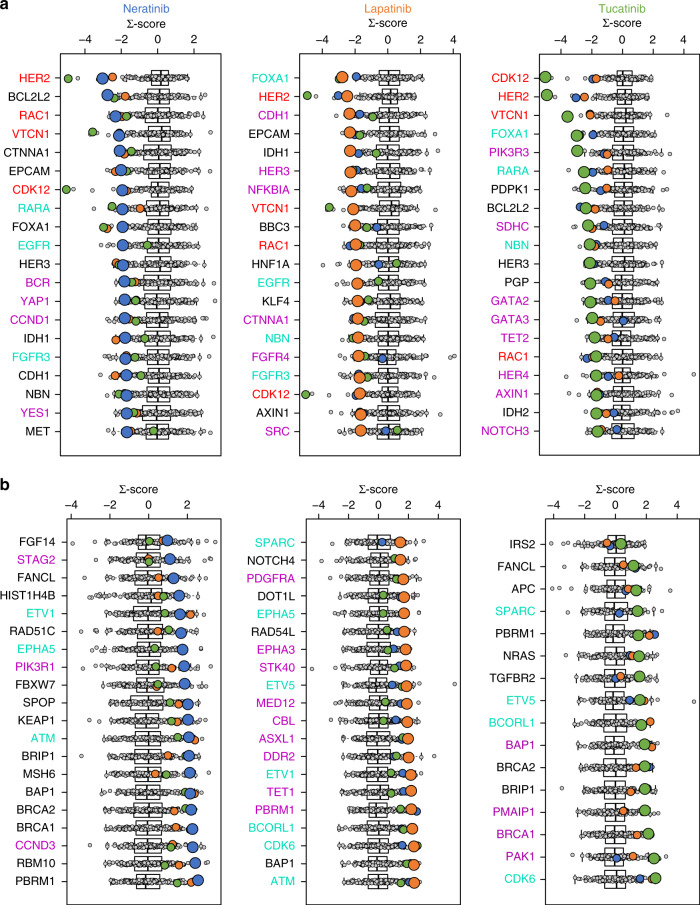
Gene expression-based biomarker analysis. Pearson correlation analysis of cell line response to Neratinib, Lapatinib, and Tucatinib with gene expression levels in 99 of 115 cancer cell lines. Correlations for each respective drug (Neratinib—blue dots, Lapatinib—orange dots, tucatinib—green dot) were compared to 168 reference compounds (grey dots). These correlations were normalised to a Σ-score. A negative score indicates a higher gene expression correlates to sensitivity to the drug. A positive score represents high gene expression correlates to insensitivity. The top 20 clinically actionable genes significantly associated with **a** sensitivity and **b** resistance are shown. Correlations of the CCLE microarray gene expression data set were validated using CCLE RNAseq, COSMIC, and Genentech mRNA expression databases. Non-black genes are considered validated, i.e. significantly correlated in at least two out of three additional gene expression data sets. In addition, genes in red and light green are validated for three out of three and two out of three HER2 TKIs, respectively. Genes in purple are validated only for the corresponding compound.

*HER2* expression correlated with sensitivity to all three TKIs and high *EGFR* levels were associated with the two EGFR-targeting TKIs, lapatinib and neratinib (Fig. 5). In addition, expression of *VTCN1*, *CDK12*, and *RAC1* correlated with sensitivity to all three compounds in the analysis, within all three independent data sets. Furthermore, several potentially actionable markers of resistance were also found. High levels of *CDK6* correlated with resistance to tucatinib and lapatinib. High expression levels of DNA repair-associated genes such as *ATM*, *BRCA1*, and *BRCA2* correlated with insensitivity to neratinib. *BRCA2* expression was also associated with tucatinib resistance.

Expression of individual genes might not fully explain the TKI cellular inhibition profiles. GSEA was therefore performed to relate drug response to expression of >3200 gene sets from the MSigDB Chemical and Genetic Perturbations collection (Supplementary Table S4). GSEA on the pre-ranked list of 18,900 correlations revealed large overlap in gene sets, which were strongly associated with drug response for all three compounds. As expected, genes upregulated in the HER2+ breast cancer subtype were strongly related to sensitivity for all three TKIs. In addition, strong correlation of various gene sets related to molecular tumour subtypes suggest that the three compounds are less effective in mesenchymal-like tumours.

## Discussion

This study aimed to compare the anti-proliferative efficacy of lapatinib, neratinib, and tucatinib in a large cancer cell line panel and to identify possible biomarkers of response or resistance to each of these HER2-targeting inhibitors. The three TKIs share several key biochemical properties but are distinct due to the nature of their binding (neratinib is an irreversible TKI; lapatinib and tucatinib are reversible inhibitors) and the affinity of neratinib (EGFR and HER4) and lapatinib (EGFR) for HER family members other than HER2.^13,31^ The three TKIs also have varying side effect profiles, most notably due to on-target EGFR inhibition, which causes higher frequency of gastrointestinal adverse events.^32^ Previous studies have shown that neratinib has greater activity than lapatinib in *HER2*-amplified and *HER2*-mutant models.^6,15^ Our study confirms this finding across multiple cancer types (Fig. 1) and provides data showing that neratinib is more effective than tucatinib in *HER2*-amplified breast cancer cell lines and in HER-altered cancer cell lines regardless of tumour tissue type (Supplementary Table S1). Biomarker analysis identified potential markers of response and resistance to all three TKIs.

Tucatinib is the most recently approved of the three TKIs and there are limited data comparing all three compounds in vitro. This study is the first comparison of the three HER2-targeted TKIs in 115 cancer cell lines. Three recent studies have carried out smaller-scale direct comparisons between neratinib, lapatinib, and tucatinib in cell line models. Nagpal et al. compared the anti-proliferative effects of neratinib, afatinib, lapatinib, and tucatinib and found that neratinib was most effective against the HER2+ human breast cancer cell line SKBR3 and the mouse-derived HER2+ cell line TBCP-1, followed by tucatinib.^33^ Brasó-Maristany et al. found that neratinib was more potent than lapatinib or tucatinib in the (HER2+ oestrogen receptor-negative (ER−)) SKBR3 and (HER2+ER+) BT474 breast cancer cell lines.^34^ The study also showed that the addition of trastuzumab enhanced the efficacy of all three TKIs. Similarly, Li et al. examined the three TKIs in combination with T-DM1.^35^ Co-treatment with neratinib and T-DM1 in *HER2*-amplified or *HER2*-mutated cell lines enhanced HER2 ubiquitination and internalisation, thereby increasing T-DM1 activity. This seems to be specific to the irreversible inhibitor, as the addition of lapatinib or tucatinib to T-DM1 did not increase HER2 ubiquitination in vitro.^34^

The HER2+ breast cancer cell lines in our study were sensitive to all three TKIs, with *HER2* amplification resulting in the strongest correlation with response in all the analyses carried out (Fig. 4). Neratinib was the most potent growth inhibitor in HER2+ breast cancer cell lines, and in the panel as a whole, while tucatinib was more effective than lapatinib in four of the five HER2+ breast cancer cell lines. The three breast cancer cell lines that showed the greatest sensitivity to the HER2-targeted TKIs (SKBR3, BT474, AU-565) have also previously shown significant in vitro trastuzumab sensitivity.^36^ MDA-MB-453 was the least TKI-responsive HER2+ breast cancer cell line. Historically, the HER2+ status of MDA-MB-453 is based on *HER2* overexpression but MDA-MB-453 is not *HER2*-amplified, as we have confirmed (Supplementary Table S3). MDA-MB-453 cells overexpress androgen receptor and could also be characterised as an apocrine subtype breast cancer cell line, perhaps partly explaining the relative insensitivity of this cell line to HER2-targeted TKIs.^37^

The genetic mutation analysis only included *HER* mutations that have previously demonstrated oncogenic potential in preclinical or clinical investigations. There may therefore be mutations relevant to response to the three studied TKIs that are present in the cell lines but were not included in the analysis. *HER4* mutations, for instance, were not included in the analyses due to a lack of validated oncogenic activity. However, two HER4 mutations (L1227M and I1226T) were present in C-33A, a cervical cancer cell line without amplification or mutations in *EGFR*, *HER2*, or *HER3*,^38^ that was potently inhibited by neratinib (Supplementary Table S1). HER4 mutations are prevalent in melanoma (4.3%), oesophagogastric (4.4%), and endometrial (3.2%) cancers and may warrant investigation as potential biomarkers of response to pan-HER TKIs.^39^

Three of the HER2 mutations in the cell line panel (S310F, R678Q, V842I) occur in known HER2 hotspots associated with sensitivity to HER2-targeted agents.^15^ The T798I mutation present in two cell lines is a ‘gatekeeper’ mutation that is reported to confer resistance to neratinib.^40^ Against this background, neratinib displayed nanomolar anti-proliferative activity against all *HER2*-mutant cell lines tested, proving more potent than lapatinib or tucatinib. Tucatinib has not yet been examined clinically against *HER2*-mutated cancers and lapatinib showed poor efficacy in a small basket trial (0/8 *HER2*-mutant NSCLC patients responded).^41^ The SUMMIT basket trial investigated neratinib in patients with solid tumours that had a *HER2* or *HER3* mutation.^42^ Of the 141 patients in this trial, encompassing 21 different cancer types, 125 had *HER2* mutations and 16 had *HER3* mutations. The SUMMIT trial provided clinical evidence (32% overall response rate at 8 weeks in breast cancer) that neratinib has actionability against several oncogenic *HER2* mutations.^42^ Neratinib and lapatinib response significantly correlated with *EGFR* mutation (Table 1 and Fig. 4). Neratinib has shown preclinical efficacy against *EGFR*-mutant models; however, previous investigations have been limited.^43^ Our results suggest that *EGFR* mutation is a better biomarker of response to TKIs than *EGFR* amplification (Table 1), which is supported by clinical observations.^44^ Within the five cell lines with mutations in *HER3* but no other family members, neratinib proved the most potent inhibitor (Table 1 and Fig. 4). *HER3* was a marker of response to neratinib and tucatinib (Fig. 4b); however, *HER3* mutations did not correlate with any TKI response in ANOVA analysis (Fig. 4a).

Beyond the HER family, this study highlights several potential novel mutation and gene expression markers of TKI response and resistance. Mutations in two chromatin re-modellers that epigenetically regulate gene expression correlated with sensitivity to lapatinib and tucatinib (*PBRM1* and *SMARCA4*, respectively; Fig. 4a). These two genes are frequently mutated in cancers, with *SMARCA4* mutations prevalent in small cell ovarian cancer and *PBRM1* altered in renal cell carcinoma.^45^ *SMARCA4*-mutated cancers may depend on SMARCA2 and are therefore susceptible to synthetic lethality with SMARCA2 inhibition. This may be a potential combinatorial strategy to further enhance tucatinib response. Our analysis also suggested that *APC* mutations are a marker of tucatinib resistance. *APC* mutations are most commonly associated with the initiation of colorectal cancer, but *APC* is also sporadically mutated or lost in breast cancer, resulting in activation of the Wnt pathway, loss of cellular polarity, and cell migration.^46^ *SMC1A* alterations were correlated with neratinib response. *SMC1A* mutations and overexpression have previously been associated with colorectal cancer aggression.^47^

The expression of four genes positively correlated with response to all three TKIs and may be markers of response to HER2-targeted TKIs: *HER2*, *RAC1*, *CDK12*, and *VTCN1* (Fig. 4a). *VTCN1*, *CDK12*, and *RAC1* represent three novel markers of HER2-targeted TKI sensitivity. *VTCN1* encodes the protein B7-H4 and has been previously associated with immunotherapy response in breast cancer, particularly in HER2+ breast cancer.^48^ CDK12 has been shown to induce trastuzumab resistance and stimulate HER2 signalling.^49^ Likewise, RAC1 has been implicated in resistance to chemotherapy, radiotherapy, and targeted therapies, such as trastuzumab.^50^ This may suggest that HER2-targeted TKIs, alone or in combination with CDK12 or RAC1 inhibition, could be a more appropriate therapeutic strategy than trastuzumab for high CDK12- or RAC1-expressing HER2+ breast cancer.

None of the genes examined in the expression analysis were associated with resistance to all three TKIs (Fig. 5b). *CDK6* expression was significantly correlated with both lapatinib and tucatinib resistance. Three CDK4/6 inhibitors are approved for the treatment of oestrogen receptor-positive (ER+) breast cancer and our results support further preclinical examination of a CDK4/6 inhibitor in combination with lapatinib or tucatinib. High expression of several DNA damage repair genes, including *ATM*, *BRCA1*, and *BRCA2*, was associated with TKI insensitivity. In addition, inactivating *BRCA2* mutations correlated with response to both neratinib and tucatinib (Fig. 4b). The addition of olaparib has been shown to enhance the effect of neratinib in triple-negative breast cancer cell line models and niraparib enhanced neratinib effectiveness in ovarian cancers.^51,52^ This suggests that TKI/poly (ADP-ribose) polymerase inhibitors may be a promising combinatorial strategy in this setting.

## Conclusions

This study provides comprehensive profiling of three HER2-targeted TKIs in a large panel of cancer cell lines. Neratinib displayed the greatest anti-proliferative activity against *HER2*-mutant and *EGFR*-mutant models compared to the other HER2-targeted TKIs and was the most potent of the three TKIs in breast cancer models. Tucatinib showed excellent selectivity for *HER2*-amplified cell lines; however, it had minimal effect on *HER2*-mutant cell lines. A list of genetic markers of response and resistance were identified for each TKI that confirm the specificity of the drugs for their targets and provide new avenues of investigation regarding potential combination strategies.

## Supplementary information

Supplemental Material

## Data Availability

The sensitivity data in this study are included in the manuscript as Supplementary Table S1. Genomic and gene expression data used in this study were gathered from publicly available databases, as described in ‘Methods’.

## References

[1] Slamon D, Godolphin W, Jones L, Holt J, Wong S, Keith D (1987). Studies of the HER-2/neu proto-oncogene in human breast and ovarian cancer. Science.

[2] Pahuja KB, Nguyen TT, Jaiswal BS, Bueno R, Jura N, Correspondence SS (2018). Actionable activating oncogenic ERBB2/HER2 transmembrane and juxtamembrane domain mutations. Cancer Cell.

[3] Zehir A, Benayed R, Shah RH, Syed A, Middha S, Kim HR (2017). Mutational landscape of metastatic cancer revealed from prospective clinical sequencing of 10,000 patients. Nat. Med..

[4] Escrivá-de-Romaní S, Arumí M, Bellet M, Saura C (2018). HER2-positive breast cancer: current and new therapeutic strategies. Breast.

[5] Keam SJ (2020). Trastuzumab deruxtecan: first approval. Drugs.

[6] Collins DM, Conlon NT, Kannan S, Verma CS, Eli LD, Lalani AS (2019). Preclinical characteristics of the irreversible pan-HER kinase inhibitor neratinib compared with lapatinib: implications for the treatment of HER2-positive and HER2-mutated breast cancer. Cancers.

[7] Murthy RK, Loi S, Okines A, Paplomata E, Hamilton E, Hurvitz SA (2020). Tucatinib, trastuzumab, and capecitabine for HER2-positive metastatic breast cancer. N. Engl. J. Med..

[8] von Minckwitz G, Procter M, de Azambuja E, Zardavas D, Benyunes M, Viale G (2017). Adjuvant pertuzumab and trastuzumab in early HER2-positive breast cancer. N. Engl. J. Med..

[9] Ryan Q, Ibrahim A, Cohen MH, Johnson J, Ko C, Sridhara R (2008). FDA drug approval summary: lapatinib in combination with capecitabine for previously treated metastatic breast cancer that overexpresses HER-2. Oncologist.

[10] Singh H, Walker AJ, Amiri-Kordestani L, Cheng J, Tang S, Balcazar P (2018). U.S. Food and Drug Administration approval: neratinib for the extended adjuvant treatment of early-stage HER2-positive breast cancer. Clin. Cancer Res..

[11] US Food & Drug Administration. FDA approves neratinib for metastatic HER2-positive breast cancer. https://www.fda.gov/drugs/resources-information-approved-drugs/fda-approves-neratinib-metastatic-her2-positive-breast-cancer (2020).

[12] US Food & Drug Administration. FDA approves tucatinib for patients with HER2-positive metastatic breast cancer. https://www.fda.gov/drugs/resources-information-approved-drugs/fda-approves-tucatinib-patients-her2-positive-metastatic-breast-cancer (2020).

[13] Kulukian A, Lee P, Taylor J, Rosler R, de Vries P, Watson D (2020). Preclinical activity of HER2-selective tyrosine kinase inhibitor tucatinib as a single agent or in combination with trastuzumab or docetaxel in solid tumor models. Mol. Cancer Ther..

[14] Zardavas D, Irrthum A, Swanton C, Piccart M (2015). Clinical management of breast cancer heterogeneity. Nat. Rev. Clin. Oncol..

[15] Gaibar M, Beltran L, Romerio-Lorca A, Fernández-Santander A, Novillo A (2020). Somatic mutations in HER2 and implications for current treatment paradigms in HER2-positive breast cancer. J. Oncol..

[16] Uitdehaag JCM, de Roos JADM, Prinsen MBW, Willemsen-Seegers N, de Vetter JRF, Dylus J (2016). Cell panel profiling reveals conserved therapeutic clusters and differentiates the mechanism of action of different PI3K/mTOR, aurora kinase and EZH2 inhibitors. Mol. Cancer Ther..

[17] Barretina J, Caponigro G, Stransky N, Venkatesan K, Margolin AA, Kim S (2012). The Cancer Cell Line Encyclopedia enables predictive modelling of anticancer drug sensitivity. Nature.

[18] Uitdehaag JCM, Kooijman JJ, de Roos JADM, MBW Prinsen, Dylus J, Willemsen-Seegers N (2019). Combined cellular and biochemical profiling to identify predictive drug response biomarkers for kinase inhibitors approved for clinical use between 2013 and 2017. Mol. Cancer Ther..

[19] Bairoch, A. The Cellosaurus, a cell-line knowledge resource. *J. Biomol. Tech.* **29**, 25–38 (2018). 10.7171/jbt.18-2902-002 PMC5945021 29805321

[20] Sveen A, Bruun J, Eide PW, Eilertsen IA, Ramirez L, Murumagi A (2018). Colorectal cancer consensus molecular subtypes translated to preclinical models uncover potentially targetable cancer cell dependencies. Clin. Cancer Res..

[21] Rees MG, Seashore-Ludlow B, Cheah JH, Adams DJ, Price EV, Gill S (2016). Correlating chemical sensitivity and basal gene expression reveals mechanism of action. Nat. Chem. Biol..

[22] Klijn C, Durinck S, Stawiski EW, Haverty PM, Jiang Z, Liu H (2015). A comprehensive transcriptional portrait of human cancer cell lines. Nat. Biotechnol..

[23] Subramanian A, Tamayo P, Mootha VK, Mukherjee S, Ebert BL, Gillette MA (2005). Gene Set Enrichment Analysis: a knowledge-based approach for interpreting genome-wide expression profiles. Proc. Natl Acad. Sci. USA.

[24] Uitdehaag, J. C. M., de Roos, J. A. D. M., van Doornmalen, A. M., Prinsen, M. B. W., Spijkers-Hagelstein, J. A. P., de Vetter, J. R. F. et al. Selective targeting of CTNNB1-, KRAS- or MYC-driven cell growth by combinations of existing drugs. *PLoS ONE* **10**, e0125021 (2015). 10.1371/journal.pone.0125021 PMC4446296 26018524

[25] Bertran-Alamillo, J., Cattan, V., Schoumacher, M., Codony-Servat, J., Giménez-Capitán, A., Cantero, F. et al. AURKB as a target in non-small cell lung cancer with acquired resistance to anti-EGFR therapy. *Nat. Commun.* **10**, 1812 (2019). 10.1038/s41467-019-09734-5 PMC6472415 31000705

[26] Vermeulen SJ, Chen TR, Speleman F, Nollet F, Van Roy FM, Mareel MM (1998). Did the four human cancer cell lines DLD-1, HCT-15, HCT-8, and HRT-18 originate from one and the same patient?. Cancer Genet. Cytogenet..

[27] Ghandi M, Huang FW, Jané-Valbuena J, Kryukov GV, Lo CC, McDonald ER (2019). Next-generation characterization of the Cancer Cell Line Encyclopedia. Nature.

[28] Ng, P. K.-S., Li, J., Jeong, K. J., Shao, S., Chen, H., Tsang, Y. H. et al. Systematic functional annotation of somatic mutations in cancer. *Cancer Cell* **33**, 450.e10–462.e10 (2018). 10.1016/j.ccell.2018.01.021 PMC5926201 29533785

[29] Krishna BM, Chaudhary S, Panda AK, Mishra DR, Mishra SK (2018). Her2 Ile655Val polymorphism and its association with breast cancer risk: an updated meta-analysis of case-control studies. Sci. Rep..

[30] Canonici A, Ivers L, Conlon NT, Pedersen K, Gaynor N, Browne BC (2018). HER-targeted tyrosine kinase inhibitors enhance response to trastuzumab and pertuzumab in HER2-positive breast cancer. Investig. N. Drugs.

[31] Collins DM, Gately K, Hughes C, Edwards C, Davies A, Madden SF (2017). Tyrosine kinase inhibitors as modulators of trastuzumab-mediated antibody-dependent cell-mediated cytotoxicity in breast cancer cell lines. Cell Immunol..

[32] Saura C, Oliveira M, Feng Y-H, Dai M-S, Hurvitz SA, Kim S-B (2019). Neratinib + capecitabine versus lapatinib + capecitabine in patients with HER2+ metastatic breast cancer previously treated with ≥ 2 HER2-directed regimens: findings from the multinational, randomized, phase III NALA trial. J. Clin. Oncol..

[33] Nagpal A, Redvers RP, Ling X, Ayton S, Fuentes M, Tavancheh E (2019). Neoadjuvant neratinib promotes ferroptosis and inhibits brain metastasis in a novel syngeneic model of spontaneous HER2+ve breast cancer metastasis. Breast Cancer Res..

[34] Brasó-Maristany F, Griguolo G, Pascual T, Paré L, Nuciforo P, Llombart-Cussac A (2020). Phenotypic changes of HER2-positive breast cancer during and after dual HER2 blockade. Nat. Commun..

[35] Li BT, Michelini F, Misale S, Cocco E, Baldino L, Cai Y (2020). HER2-mediated internalization of cytotoxic agents in *ERBB2* amplified or mutant lung cancers. Cancer Discov..

[36] O’Brien NA, Browne BC, Chow L, Wang Y, Ginther C, Arboleda J (2010). Activated phosphoinositide 3-kinase/AKT signaling confers resistance to trastuzumab but not lapatinib. Mol. Cancer Ther..

[37] Moore NL, Buchanan G, Harris JM, Selth LA, Bianco-Miotto T, Hanson AR (2012). An androgen receptor mutation in the MDA-MB-453 cell line model of molecular apocrine breast cancer compromises receptor activity. Endocr. Relat. Cancer.

[38] Meira DD, de Almeida VH, Mororó JS, Nóbrega I, Bardella L, Silva RLA (2009). Combination of cetuximab with chemoradiation, trastuzumab or MAPK inhibitors: mechanisms of sensitisation of cervical cancer cells. Br. J. Cancer.

[39] Mishra R, Hanker AB, Garrett JT (2017). Genomic alterations of ERBB receptors in cancer: clinical implications. Oncotarget.

[40] Connell CM, Doherty GJ (2017). Activating HER2 mutations as emerging targets in multiple solid cancers. ESMO Open.

[41] Lopez-Chavez A, Thomas A, Rajan A, Raffeld M, Morrow B, Kelly R (2015). Molecular profiling and targeted therapy for advanced thoracic malignancies a biomarker-derived, multiarm, multihistology phase II basket trial. J. Clin. Oncol..

[42] Hyman DM, Piha-Paul SA, Won H, Rodon J, Saura C, Shapiro GI (2018). HER kinase inhibition in patients with HER2- and HER3-mutant cancers. Nature.

[43] Castellano GM, Aisner J, Burley SK, Vallat B, Yu HA, Pine SR (2019). A novel acquired exon 20 EGFR M766Q mutation in lung adenocarcinoma mediates osimertinib resistance but is sensitive to neratinib and poziotinib. J. Thorac. Oncol..

[44] Sholl LM, Xiao Y, Joshi V, Yeap BY, Cioffredi LA, Jackman DM (2010). EGFR mutation is a better predictor of response to tyrosine kinase inhibitors in non-small cell lung carcinoma than FISH, CISH, and immunohistochemistry. Am. J. Clin. Pathol..

[45] Hodges, C., Kirkland, J. G. & Crabtree, G. R. The many roles of BAF (mSWI/SNF) and PBAF complexes in cancer. *Cold Spring Harb. Perspect. Med*. **6**, a026930 (2016). 10.1101/cshperspect.a026930 PMC4968166 27413115

[46] Razavi P, Chang MT, Xu G, Bandlamudi C, Ross DS, Vasan N (2018). The genomic landscape of endocrine-resistant advanced breast cancers. Cancer Cell.

[47] Sarogni, P., Palumbo, O., Servadio, A., Astigiano, S., D’Alessio, B., Gatti, V. et al. Overexpression of the cohesin-core subunit SMC1A contributes to colorectal cancer development. *J Exp. Clin. Cancer Res.* **38**, 108 (2019). 10.1186/s13046-019-1116-0 PMC6397456 30823889

[48] Kim JY, Lee E, Park K, Park WY, Jung HH, Ahn JS (2017). Immune signature of metastatic breast cancer: Identifying predictive markers of immunotherapy response. Oncotarget.

[49] Choi, H., Jin, S., Cho, H., Won, H., An, H. W., Jeong, G. et al. CDK 12 drives breast tumor initiation and trastuzumab resistance via WNT and IRS 1‐ErbB‐ PI 3K signaling. *EMBO Rep.* **20**, e48058 (2019). 10.15252/embr.201948058 PMC6776914 31468695

[50] Zhao Y, Wang Z, Jiang Y, Yang C (2011). Inactivation of Rac1 reduces Trastuzumab resistance in PTEN deficient and insulin-like growth factor I receptor overexpressing human breast cancer SKBR3 cells. Cancer Lett..

[51] Pierce A, Mcgowan PM, Cotter M, Mullooly M, O’Donovan N, Rani S (2013). Comparative antiproliferative efects of iniparib and olaparib on a panel of triple-negative and non-triple-negative breast cancer cell lines. Cancer Biol. Ther..

[52] Booth L, Roberts JL, Samuel P, Avogadri-Connors F, Cutler RE, Lalani AS (2018). The irreversible ERBB1/2/4 inhibitor neratinib interacts with the PARP1 inhibitor niraparib to kill ovarian cancer cells. Cancer Biol. Ther..

