# Supplementary material for: Comparative analysis of drug response and gene profiling of HER2-targeted tyrosine kinase inhibitors
Source: Br J Cancer. 2021 Jan 21;124(7):1249–59. doi: 10.1038/s41416-020-01257-x (PMC8007737; doi:10.1038/s41416-020-01257-x)
Supplement: Supplementary file 1 — Supplemental Material [file 41416_2020_1257_MOESM1_ESM.docx]

**Supplementary table S1:** IC_50_ values of the HER2-targeted TKIs neratinib, lapatinib, tucatinib, dacomitinib, afatinib in 115 cancer cell lines and poziotinib and pyrotinib in the 12 breast cancer cell lines. The cell lines included in each dataset of the gene expression and clustering analyses are indicated by dots.

|  |  |  |  |  |  |  |  |  |  | **Clustering analysis** | **Gene expression analysis** | | | |
| --- | --- | --- | --- | --- | --- | --- | --- | --- | --- | --- | --- | --- | --- | --- |
| **Cell line** | **Supplier ref** | **Disease** | **Neratinib** | **Lapatinib** | **Tucatinib** | **Dacomitinib** | **Afatinib** | **Poziotinib** | **Pyrotinib** |  | **CCLE microarray** | **CCLE RNAseq** | **Genentech** | **COSMIC** |
| **5637** | HTB-9 | Bladder carcinoma | 405 | 2220 | 8877 | 31 | 16 |  |  | ● | ● | ● |  | ● |
| **769-P** | CRL-1933 | Renal cell carcinoma | 990 | 176 | 20220 | 32 | 1802 |  |  | ● | ● | ● | ● | ● |
| **786-O** | CRL-1932 | Renal cell carcinoma | 1225 | 10507 | 21285 | 3039 | 2218 |  |  | ● | ● | ● | ● | ● |
| **A-172** | CRL-1620 | Glioblastoma | 2274 | 13738 | 17392 | 3621 | 2645 |  |  | ● | ● | ● | ● | ● |
| **A-204** | HTB-82 | Embryonal rhabdomyosarcoma | 555 | 10814 | 11434 | 2508 | 1650 |  |  | ● | ● | ● |  | ● |
| **A375** | CRL-1619 | Amelanotic melanoma | 1420 | 19743 | 11270 | 3460 | 2454 |  |  | ● | ● | ● | ● | ● |
| **A388** | CRL-7905 | Squamous cell carcinoma | 143 | 1201 | 15920 | 19 | 195 |  |  | ● |  |  |  | ● |
| **A-427** | HTB-53 | Lung adenocarcinoma | 1125 | 12693 | 22240 | 2892 | 2797 |  |  | ● |  | ● | ● | ● |
| **A-498** | HTB-44 | Renal cell carcinoma | 886 | 261 | 20445 | 2569 | 8.7 |  |  | ● | ● | ● | ● | ● |
| **A-549** | CCL-185 | Lung adenocarcinoma | 2247 | 675 | 25690 | 6634 | 4300 |  |  | ● | ● | ● | ● | ● |
| **A-704** | HTB-45 | Renal cell carcinoma | 1590 | 26486 | 31600 | 3905 | 4815 |  |  | ● | ● | ● |  | ● |
| **ACHN** | CRL-1611 | Papillary renal cell carcinoma | 406 | 304 | 13873 | 11 | 4.1 |  |  | ● | ● | ● | ● | ● |
| **AN3 CA** | HTB-111 | Endometrial adenocarcinoma | 2147 | 12846 | 24528 | 3746 | 3158 |  |  | ● | ● | ● | ● | ● |
| **AsPC-1** | CRL-1682 | Pancreatic ductal adenocarcinoma | 1387 | 12793 | 31600 | 5823 | 405 |  |  | ● | ● | ● | ● | ● |
| **AU-565** | CRL-2351 | Breast adenocarcinoma | 20 | 294 | 125 | 68 | 27 | 1.8 | 20 | ● | ● | ● |  | ● |
| **BT-20** | HTB-19 | Invasive ductal carcinoma | 4303 | 15208 | 28834 | 5622 | 4104 | 24577 | 3160 | ● | ● | ● | ● | ● |
| **BT-474** | HTB-20 | Breast ductal carcinoma | 59 | 262 | 29 | 37 | 7.0 | 1.1 | 5.4 |  | ● | ● | ● | ● |
| **BT-549** | HTB-122 | Invasive ductal carcinoma | 2080 | 11520 | 15903 | 3678 | 1817 | 5119 | 515 | ● | ● | ● | ● | ● |
| **BxPC-3** | CRL-1687 | Pancreatic ductal adenocarcinoma | 236 | 11998 | 12580 | 4064 | 26 |  |  | ● | ● | ● | ● | ● |
| **C-33 A** | HTB-31 | Cervical squamous cell carcinoma | 49 | 258 | 412 | 40 | 36 |  |  | ● |  | ● | ● | ● |
| **CAL 27** | CRL-2095 | Tongue squamous cell carcinoma | 130 | 879 | 13660 | 11 | 6.0 |  |  | ● | ● | ● | ● | ● |
| **CCF-STTG1** | CRL-1718 | Astrocytoma | 3933 | 22514 | 31600 | 5610 | 5600 |  |  | ● | ● | ● |  | ● |
| **CCRF-CEM** | CCL-119 | Childhood T acute lymphoblastic leukemia | 2415 | 11159 | 17676 | 3266 | 1119 |  |  | ● |  |  |  | ● |
| **COLO 205** | CCL-222 | Colon adenocarcinoma | 2196 | 17026 | 22213 | 4279 | 2056 |  |  | ● | ● |  | ● | ● |
| **COLO 829** | CRL-1974 | Cutaneous melanoma | 3688 | 13206 | 20569 | 6023 | 4575 |  |  | ● | ● | ● | ● | ● |
| **Daoy** | HTB-186 | Medulloblastoma | 803 | 18384 | 30244 | 3767 | 2633 |  |  | ● | ● | ● |  | ● |
| **DB** | CRL-2289 | Diffuse large B-cell lymphoma | 1705 | 12375 | 19192 | 3596 | 741 |  |  | ● | ● | ● | ● | ● |
| **DLD-1** | CCL-221 | Colon adenocarcinoma | 2035 | 14634 | 23218 | 3240 | 2382 |  |  | ● | ● | ● | ● |  |
| **DoTc2 4510** | CRL-7920 | Cervical carcinoma | 42 | 341 | 5591 | 12 | 3.7 |  |  | ● |  | ● |  | ● |
| **DU 145** | HTB-81 | Prostate carcinoma | 198 | 530 | 6879 | 18 | 16 |  |  | ● | ● | ● | ● | ● |
| **DU4475** | HTB-123 | Breast carcinoma | 3968 | 5835 | 10658 | 5242 | 4126 | 8305 | 3160 | ● | ● | ● | ● | ● |
| **ES-2** | CRL-1978 | Ovarian clear cell adenocarcinoma | 702 | 12027 | 18355 | 2994 | 852 |  |  | ● | ● | ● | ● | ● |
| **FaDu** | HTB-43 | Hypopharyngeal squamous cell carcinoma | 216 | 245 | 14075 | 13 | 2.9 |  |  | ● | ● | ● | ● | ● |
| **G-361** | CRL-1424 | Melanoma | 719 | 11027 | 13477 | 4113 | 2028 |  |  | ● | ● | ● | ● | ● |
| **HCC1954** | CRL-2338 | Ductal breast carcinoma | 138 | 1426 | 2122 | 10 | 2.8 | 0.6 | 29 |  | ● | ● | ● | ● |
| **HCT 116** | CCL-247 | Colon carcinoma | 867 | 13728 | 21924 | 2304 | 1113 |  |  | Yes | ● | ● | ● | ● |
| **HCT-15** | CCL-225 | Colon adenocarcinoma | 2078 | 11829 | 16660 | 3446 | 1872 |  |  | Yes | ● | ● | ● | ● |
| **HEC-1** | JCRB0042 | Endometrial adenocarcinoma | 84 | 31600 | 13943 | 1911 | 1800 |  |  |  |  |  |  | ● |
| **HEC-1-B** | HTB-113 | Endometrial adenocarcinoma | 100 | 31600 | 1620 | 14 | 459 |  |  |  | ● | ● |  |  |
| **HEC-251** | JCRB1141 | Endometrial carcinoma | 602 | 1073 | 17286 | 29 | 1786 |  |  |  | ● | ● |  |  |
| **HEC-6** | JCRB1118 | Endometrial endometrioid adenocarcinoma | 166 | 6201 | 14810 | 2511 | 2978 |  |  |  | ● | ● |  |  |
| **HL-60** | CCL-240 | Adult acute myeloid leukemia | 1459 | 13463 | 16937 | 1737 | 2016 |  |  | ● | ● | ● | ● | ● |
| **Hs 578T** | HTB-126 | Invasive ductal carcinoma | 1467 | 15207 | 24107 | 3094 | 2526 | 6384 | 1890 | ● | ● | ● | ● | ● |
| **Hs 746T** | HTB-135 | Gastric adenocarcinoma | 2516 | 13426 | 12440 | 3119 | 3268 |  |  | ● | ● | ● | ● | ● |
| **Hs 766T** | HTB-134 | Pancreatic adenocarcinoma | 2185 | 17135 | 31600 | 5007 | 4159 |  |  | ● | ● | ● | ● | ● |
| **HT** | CRL-2260 | Diffuse large B-cell lymphoma | 10000 | 13149 | 29876 | 3764 | 860 |  |  | ● | ● | ● | ● |  |
| **HT-1080** | CCL-121 | Fibrosarcoma | 816 | 10728 | 17150 | 2789 | 2541 |  |  | ● | ● | ● | ● | ● |
| **HuTu 80** | HTB-40 | Duodenal adenocarcinoma | 1673 | 12784 | 623 | 4064 | 3524 |  |  | ● | ● | ● |  | ● |
| **II-18** | RCB2093 | Lung adenocarcinoma | 123 | 10410 | 825 | 2.0 | 1.1 |  |  |  |  |  |  |  |
| **J82** | HTB-1 | Bladder carcinoma | 2399 | 18157 | 31600 | 3948 | 3741 |  |  | ● | ● | ● |  | ● |
| **JAR** | HTB-144 | Gestational choriocarcinoma | 1169 | 10960 | 24143 | 3052 | 1917 |  |  | ● |  | ● |  | ● |
| **Jurkat E6.1** | TIB-152 | Childhood T acute lymphoblastic leukemia | 1421 | 10548 | 14748 | 3183 | 1808 |  |  | ● | ● | ● | ● | ● |
| **K-562** | CCL-243 | Chronic myelogenous leukemia | 889 | 12217 | 8528 | 3435 | 2721 |  |  | ● | ● | ● | ● | ● |
| **KATO III** | HTB-103 | Signet ring cell gastric adenocarcinoma | 826 | 10940 | 17882 | 2961 | 1910 |  |  | ● | ● | ● | ● | ● |
| **KG-1** | CCL-246 | Adult acute myeloid leukemia | 488 | 18543 | 16177 | 2567 | 3239 |  |  | ● | ● | ● | ● | ● |
| **KLE** | CRL-1622 | Endometrial adenocarcinoma | 106 | 16661 | 4737 | 3437 | 0.8 |  |  | ● | ● | ● | ● | ● |
| **KU812** | CRL-2099 | Chronic myelogenous leukemia | 2614 | 12352 | 22634 | 3195 | 1394 |  |  | ● | ● | ● | ● | ● |
| **LNCaP FGC** | CRL-1740 | Prostate carcinoma | 2867 | 21069 | 21813 | 7972 | 7109 |  |  | ● | ● | ● | ● | ● |
| **LoVo** | CCL-229 | Colon adenocarcinoma | 1809 | 338 | 25729 | 4301 | 2309 |  |  | ● | ● | ● | ● | ● |
| **LS 174T** | CL-188 | Colon adenocarcinoma | 2482 | 109 | 20252 | 2669 | 89 |  |  | ● |  |  | ● |  |
| **LS411N** | CRL-2159 | Cecum adenocarcinoma | 4085 | 18008 | 21392 | 4240 | 1150 |  |  | ● | ● | ● |  | ● |
| **MCF7** | HTB-22 | Invasive ductal carcinoma | 10000 | 18892 | 30520 | 12912 | 11880 | 31600 | 3160 | ● | ● | ● | ● | ● |
| **MDA-MB-231** | HTB-26 | breast adenocarcinoma | 4127 | 10000 | 22473 | 3691 | 1895 | 7023 | 2086 |  | ● | ● | ● | ● |
| **MDA-MB-453** | HTB-131 | breast carcinoma | 3062 | 2844 | 5928 | 3249 | 182 | 6.1 | 69 |  | ● | ● | ● | ● |
| **MDA-MB-468** | HTB-132 | breast adenocarcinoma | 1539 | 10000 | 8881 | 33 | 25 | 5.9 | 508 |  | ● | ● | ● | ● |
| **MeWo** | HTB-65 | Melanoma | 2830 | 15527 | 31600 | 3917 | 4279 |  |  | ● | ● | ● | ● | ● |
| **MG-63** | CRL-1427 | Osteosarcoma | 3333 | 13191 | 29446 | 4003 | 3339 |  |  | ● | ● | ● | ● | ● |
| **MIA PaCa-2** | CRL-1420 | Pancreatic ductal adenocarcinoma | 2889 | 16957 | 18260 | 3932 | 2292 |  |  | ● | ● | ● | ● | ● |
| **MOLT-4** | CRL-1582 | Adult T acute lymphoblastic leukemia | 2375 | 11432 | 20268 | 3643 | 1264 |  |  | ● | ● |  |  | ● |
| **NCCIT** | CRL-2073 | Testicular embryonal carcinoma | 1684 | 18075 | 31600 | 4434 | 1753 |  |  | ● |  |  |  | ● |
| **NCI-H1975** | CRL-5908 | Lung adenocarcinoma | 79 | 8813 | 24517 | 153 | 106 |  |  |  | ● | ● | ● | ● |
| **NCI-H460** | HTB-177 | Large cell lung carcinoma | 2759 | 22342 | 30374 | 4685 | 2933 |  |  | ● | ● | ● | ● |  |
| **NCI-H661** | HTB-183 | Large cell lung carcinoma | 2017 | 15208 | 26885 | 3683 | 12 |  |  | ● | ● | ● |  | ● |
| **NCI-H82** | HTB-175 | Small cell lung carcinoma | 2009 | 12443 | 20436 | 3907 | 2679 |  |  | ● | ● | ● | ● | ● |
| **OVCAR-3** | HTB-161 | High grade ovarian serous adenocarcinoma | 1351 | 31600 | 31600 | 23 | 1309 |  |  | ● | ● | ● | ● | ● |
| **PA-1** | CRL-1572 | Ovarian mixed germ cell tumor | 603 | 11144 | 21058 | 2697 | 2597 |  |  | ● |  | ● | ● | ● |
| **PC-3** | CRL-1435 | Prostate carcinoma | 5085 | 11930 | 26835 | 5257 | 4648 |  |  | ● | ● | ● | ● | ● |
| **PFSK-1** | CRL-2060 | Primitive neuroectodermal tumor | 2782 | 10957 | 19030 | 4477 | 2576 |  |  | ● |  | ● |  | ● |
| **RD** | CCL-136 | Embryonal rhabdomyosarcoma | 932 | 16624 | 28828 | 2527 | 1906 |  |  | ● | ● | ● |  | ● |
| **RKO** | CRL-2577 | Colon carcinoma | 1766 | 8227 | 13082 | 3580 | 2717 |  |  | ● | ● | ● | ● | ● |
| **RL** | CRL-2261 | Diffuse large B-cell lymphoma | 4765 | 11896 | 23968 | 4153 | 1900 |  |  | ● | ● | ● | ● | ● |
| **RL95-2** | CRL-1671 | Endometrial adenosquamous carcinoma | 172 | 110 | 1761 | 7.8 | 4.6 |  |  | ● | ● | ● |  | ● |
| **RPMI-7951** | HTB-66 | Melanoma | 2301 | 12214 | 15794 | 3969 | 3598 |  |  | ● | ● | ● | ● | ● |
| **RS4-11** | CRL-1873 | Adult B acute lymphoblastic leukemia | 1474 | 13102 | 24447 | 3357 | 2330 |  |  | ● | ● | ● |  | ● |
| **RT4** | HTB-2 | Bladder carcinoma | 1101 | 274 | 9989 | 14 | 10 |  |  | ● | ● | ● | ● | ● |
| **SHP-77** | CRL-2195 | Small cell lung carcinoma | 4916 | 18132 | 22761 | 5075 | 3766 |  |  | ● | ● | ● |  | ● |
| **SJCRH30** | CRL-2061 | Alveolar rhabdomyosarcoma | 2362 | 12127 | 25838 | 3926 | 2507 |  |  | ● | ● |  | ● | ● |
| **SK-BR-3** | HTB-30 | Breast adenocarcinoma | 7 | 152 | 22 | 27 | 3.9 | 1.1 | 8.0 |  | ● | ● | ● |  |
| **SK-N-AS** | CRL-2137 | Neuroblastoma | 1632 | 15494 | 25207 | 3085 | 1929 |  |  | ● | ● | ● | ● | ● |
| **SK-N-FI** | CRL-2142 | Neuroblastoma | 1048 | 19991 | 31600 | 3362 | 1914 |  |  | ● | ● | ● | ● | ● |
| **SNU-5** | CRL-5973 | Gastric carcinoma | 772 | 17266 | 24581 | 3409 | 3882 |  |  | ● | ● | ● | ● | ● |
| **SNU-C2B** | CCL-250 | Cecum adenocarcinoma | 251 | 8395 | 31600 | 5590 | 265 |  |  | ● | ● |  |  | ● |
| **SR** | CRL-2262 | Anaplastic large cell lymphoma | 1745 | 3715 | 16801 | 3551 | 1208 |  |  | ● | ● | ● | ● | ● |
| **SU-DHL-1** | CRL-2955 | Anaplastic large cell lymphoma | 2647 | 6478 | 15748 | 3509 | 2844 |  |  | ● | ● | ● | ● | ● |
| **SU-DHL-6** | CRL-2959 | Diffuse large B-cell lymphoma | 1548 | 9010 | 15275 | 2547 | 862 |  |  | ● | ● | ● | ● | ● |
| **SUP-T1** | ACC140 | Childhood T lymphoblastic lymphoma | 3676 | 11760 | 31600 | 4543 | 3824 |  |  | ● | ● | ● |  | ● |
| **SW48** | CCL-231 | Colon adenocarcinoma | 9 | 97 | 17373 | 0.6 | 0.3 |  |  | ● | ● | ● | ● | ● |
| **SW480** | CCL-228 | Colon adenocarcinoma | 3374 | 13205 | 31600 | 4827 | 4547 |  |  | ● | ● | ● | ● |  |
| **SW620** | CCL-227 | Colon adenocarcinoma | 3220 | 20741 | 31600 | 5177 | 4144 |  |  | ● | ● | ● | ● | ● |
| **SW626** | HTB-78 | Colon adenocarcinoma | 634 | 12904 | 23509 | 5244 | 3840 |  |  | ● |  | ● |  | ● |
| **SW837** | CCL-235 | Rectal adenocarcinoma | 4243 | 17314 | 31600 | 6484 | 4594 |  |  | ● | ● | ● | ● | ● |
| **SW872** | HTB-92 | Liposarcoma | 3805 | 12982 | 31370 | 5030 | 4633 |  |  | ● |  |  |  | ● |
| **SW900** | HTB-59 | Squamous cell lung carcinoma | 2304 | 21519 | 31600 | 4742 | 3426 |  |  | ● | ● | ● | ● | ● |
| **SW948** | CCL-237 | Colon adenocarcinoma | 3559 | 14010 | 31600 | 4802 | 3470 |  |  | ● | ● | ● | ● | ● |
| **SW982** | HTB-93 | Biphasic synovial sarcoma | 1812 | 18496 | 31600 | 3940 | 2882 |  |  | ● |  | ● | ● | ● |
| **T24** | HTB-4 | Bladder carcinoma | 543 | 16543 | 20124 | 3025 | 2644 |  |  | ● | ● | ● |  | ● |
| **T98G** | CRL-1690 | Glioblastoma | 3211 | 22141 | 30058 | 4589 | 4990 |  |  | ● | ● | ● | ● | ● |
| **TCCSUP** | HTB-5 | Bladder carcinoma | 853 | 14001 | 30425 | 3387 | 3654 |  |  | ● | ● | ● |  | ● |
| **THP-1** | TIB-202 | Childhood acute monocytic leukemia | 967 | 19184 | 31600 | 3912 | 3228 |  |  | ● | ● | ● | ● | ● |
| **TT** | CRL-1803 | Hereditary thyroid gland medullary carcinoma | 4218 | 17479 | 31600 | 4827 | 4670 |  |  | ● | ● | ● |  | ● |
| **U-118 MG** | HTB-15 | Astrocytoma | 2078 | 20764 | 24960 | 3630 | 2958 |  |  | ● | ● | ● |  | ● |
| **U-2 OS** | HTB-96 | Osteosarcoma | 646 | 14006 | 22094 | 3312 | 2283 |  |  | ● | ● | ● | ● | ● |
| **U-87 MG** | HTB-14 | Glioblastoma | 3262 | 17195 | 31600 | 4413 | 4294 |  |  | ● | ● | ● |  | ● |
| **UM-UC-3** | CRL-1749 | Bladder carcinoma | 123 | 31600 | 8398 | 3187 | 3344 |  |  |  | ● | ● | ● | ● |
| **VA-ES-BJ** | CRL-2138 | Epithelioid sarcoma | 1790 | 20784 | 25855 | 3554 | 2526 |  |  | ● |  | ● |  | ● |

**Supplementary table S2:** Oligonucleotide sequences used in targeted sequencing of EGFR, HER2, and HER3.

| Chromosome | Gene Mutation | ULSO Sequence | DLSO Sequence | ULSO start Position | DLSO end Position |
| --- | --- | --- | --- | --- | --- |
| chr7 | EGFR_R165Q | CGACATGTTGCTGAGAAAGTCACTG | TTTCTGCGGGAGAATGGAACTGCGG | 55214277 | 55214405 |
| chr7 | EGFR_A289D_V | GGGACAAGGATGCCTGACCAGTTAG | GGGTTGTAGAGCATGAGTGGGGGGC | 55221747 | 55221893 |
| chr7 | EGFR_G719S | CCTGTGCCAGGGACCTTACCTTATA | TTCTCCACTGGGTGTAAGAGGCTCC | 55241619 | 55241755 |
| chr7 | EGFR_746-750del | ACTCACATCGAGGATTTCCTTGTTG | GAGAAGGAAGACGTTAACTGGCAAT | 55242378 | 55242519 |
| chr7 | EGFR_T790M | TCTTTGTGTTCCCGGACATAGTCCA | CATCACGTAGGCTTCCTGGAGGGAG | 55248976 | 55249122 |
| chr7 | EGFR_L858R | AGGAAAATGCTGGCTGACCTAAAGC | GTGTTTTCACCAGTACGTTCCTGGC | 55259460 | 55259603 |
| chr12 | ERBB3_M91I | GCGGAGGTTGGGCAATGGTAGAGTA | GCAAAATAATCTCAATCTCCTCTCC | 56478714 | 56478853 |
| chr12 | ERBB3_N126K | AGGGGTGAGGCCAGAAGGAACCATC | ATCGTAGACCTGGGTCCCTCGCACC | 56478856 | 56479000 |
| chr12 | ERBB3_D297H | ATTTTTATCTACTTCCATCTTGTCA | ATCACCAGCTACCAATGGAACCCTT | 56482259 | 56482397 |
| chr12 | ERBB3_R475W | CCTGACTCCCTATTCTCACCACCTA | CAAAGAGTGGTGGTAGCAGAGCTGC | 56487234 | 56487377 |
| chr12 | ERBB3_R667H | ATCGCCTCATAGCCCTTTTATTCTG | TCAGATGGGTTTTGCTATGGGATAT | 56489438 | 56489573 |
| chr12 | ERBB3_E928G | CACCATGTAGACATCAATTGTGCAG | AACTCCCAAACTGTCACACCTGTTG | 56492537 | 56492679 |
| chr12 | ERBB3_E952Q | CCTACTCACCTTTATGACCAGATAC | TGGAGATAAGATGTAGGAGGTAGAG | 56493406 | 56493538 |
| chr12 | ERBB3_P1142H | CTAGGAAGAGTCAGGCACCTTTGAG | AATGGTAGGCGCTATCTCCGCGTGG | 56495019 | 56495163 |
| chr17 | ERBB2_S310F | CCTCTGCTGTCACCTCTTGGTTGTG | ACCATGAGCAGCATTACCGTGCCCA | 37868107 | 37868255 |
| chr17 | ERBB2_R678Q | TCACCTCCGTTTCCTGCAGCAGTCT | GATGGACGTCAGAGGGCTGGGGGCG | 37879563 | 37879714 |
| chr17 | ERBB2_T798I | GCGGTTTTCCCGGACATGGTCTAAG | CATCACGTATGCTTCCTGGGGACAA | 37880969 | 37881113 |
| chr17 | ERBB2_V842I | GAGCCAGCCCGAAGTCTGTAATTTT | AAGCACCCATGTAGACCTTCTGGGA | 37881267 | 37881410 |
|  |  |  |  |  |  |
| ULSO: Upstream Locus-Specific Oligo | | |  |  |  |
| DLSO: Downstream Locus-Specific Oligo | | |  |  |  |

**Supplementary table S3:** Validation of HER mutations reported in CCLE by targeted sequencing.

|  |  |  |  | **NTRC** | | |  | **CCLE** | | |  |
| --- | --- | --- | --- | --- | --- | --- | --- | --- | --- | --- | --- |
| **Cell line** | **Gene** | **Mutation CDS** | **Mutation AA** | **Total reads** | **Alt reads** | **Alt freq** |  | **Total reads** | **Alt reads** | **Alt freq** |  |
| **5637** | ERBB2 | c.929C>T | p.S310F | 25981 | 13905 | 0.54 |  | 888 | 502 | 0.57 |  |
| **AU-565** | ERBB2 | Amplification |  |  |  |  |  |  |  |  |  |
|  | ERBB3 | c.2854G>C | p.E952Q | 10654 | 5307 | 0.50 |  | 691 | 362 | 0.52 |  |
| **DLD-1** | ERBB3 | c.3425C>A | p.P1142H | 18263 | 9078 | 0.50 |  | *No sequence data in CCLE* |  |  |  |
|  | ERBB3 | c.2000G>A | p.R667H | 20612 | 9586 | 0.47 |  | *No sequence data in CCLE* |  |  |  |
|  | ERBB3 | c.378C>A | p.N126K | 18126 | 8385 | 0.46 |  | *No sequence data in CCLE* |  |  |  |
| **FaDu** | ERBB2 | c.1963A>G | p.I655V | 38920 | 22678 | 0.58 |  | *Mutation not found* |  |  |  |
|  | ERBB3 | c.889G>C | p.D297H | 20531 | 3958 | 0.19 |  | 298 | 15 | 0.05 |  |
| **HCT-15** | ERBB3 | c.378C>A | p.N126K | 17372 | 8627 | 0.50 |  | 478 | 239 | 0.50 |  |
|  | ERBB3 | c.3425C>A | p.P1142H | 16963 | 8138 | 0.48 |  | 345 | 189 | 0.55 |  |
|  | ERBB3 | c.2000G>A | p.R667H | 29528 | 13514 | 0.46 |  | 650 | 332 | 0.51 |  |
| **HEC-1** | ERBB2 | c.1963A>G | p.I655V | 33302 | 17350 | 0.52 |  | *Mutation not found* |  |  |  |
|  | ERBB2 | c.2524G>A | p.V842I | 39473 | 19339 | 0.49 |  | 119 | 61 | 0.51 |  |
|  | ERBB2 | c.2393C>T | p.T798I | 21250 | 10377 | 0.49 |  | 94 | 51 | 0.54 |  |
|  | ERBB3 | c.1423C>T | p.R475W | 26300 | 12915 | 0.49 |  | 170 | 79 | 0.46 |  |
| **HEC-1-B** | EGFR | c.2591C>T | p.A864V | 21435 | 5002 | 0.23 |  | 237 | 62 | 0.26 |  |
|  | ERBB2 | c.1963A>G | p.I655V | 45768 | 19568 | 0.43 |  | *Mutation not found* |  |  |  |
|  | ERBB2 | c.2524G>A | p.V842I | 53438 | 21139 | 0.40 |  | 719 | 275 | 0.38 |  |
|  | ERBB2 | c.2393C>T | p.T798I | 24927 | 9643 | 0.39 |  | 1571 | 636 | 0.40 |  |
|  | ERBB3 | c.1423C>T | p.R475W | 27770 | 13504 | 0.49 |  | 67 | 27 | 0.40 |  |
| **HEC-6** | EGFR | c.866C>T | p.A289V | 8711 | 2031 | 0.23 |  | 301 | 82 | 0.27 |  |
|  | ERBB3 | c.2000G>A | p.R667H | 20428 | 5754 | 0.28 |  | 334 | 113 | 0.34 |  |
|  | ERBB3 | c.2783A>G | p.E928G | 22771 | 5187 | 0.23 |  | 189 | 12 | 0.06 |  |
| **HEC-251** | ERBB3 | c.1423C>T | p.R475W | 22922 | 11412 | 0.50 |  | 102 | 51 | 0.50 |  |
| **II-18** | EGFR | c.2573T>G | p.L858R | 20706 | 11828 | 0.57 |  | *Cell line not in CCLE* |  |  |  |
| **J82** | ERBB2 | c.1963A>G | p.I655V | 43692 | 31218 | 0.72 |  | *Mutation not found* |  |  |  |
|  | ERBB2 | c.2033G>A | p.R678Q | 43731 | 12270 | 0.28 |  | 1198 | 382 | 0.32 |  |
| **NCI-H1975** | EGFR | c.2369C>T | p.T790M | 19272 | 13308 | 0.69 |  | 545 | 398 | 0.73 |  |
|  | EGFR | c.2573T>G | p.L858R | 21090 | 14319 | 0.68 |  | 401 | 321 | 0.80 |  |
| **RD** | ERBB2 | c.1963A>G | p.I655V | 35516 | 35420 | 1.00 |  | *Mutation not found* |  |  |  |
| **RL95-2** | EGFR | c.866C>T | p.A289V | 11905 | 1475 | 0.12 |  | 527 | 80 | 0.15 |  |
| **SK-BR-3** | ERBB2 | Amplification |  |  |  |  |  |  |  |  |  |
|  | ERBB3 | c.2854G>C | p.E952Q | 10927 | 5251 | 0.48 |  | 481 | 250 | 0.52 |  |
| **SNU-C2B** | EGFR | c.494G>A | p.R165Q | 33704 | 17339 | 0.51 |  | 19 | 8 | 0.42 |  |
|  | ERBB2 | c.2033G>A | p.R678Q | 31546 | 14966 | 0.48 |  | 67 | 32 | 0.48 |  |
| **SW48** | ERBB2 | c.1963A>G | p.I655V | 35394 | 17912 | 0.51 |  | *Mutation not found* |  |  |  |
|  | EGFR | c.2155G>A | p.G719S | 32157 | 10639 | 0.33 |  | 943 | 364 | 0.39 |  |
| **UM-UC-3** | ERBB3 | c.273G>A | p.M91I | 38638 | 22924 | 0.59 |  | 137 | 75 | 0.55 |  |
| **A388** | EGFR | Amplification |  |  |  |  |  |  |  |  |  |
| **BT-20** | EGFR | Amplification |  |  |  |  |  |  |  |  |  |
| **BT-474** | ERBB2 | Amplification |  |  |  |  |  |  |  |  |  |
| **HCC1954** | ERBB2 | Amplification |  |  |  |  |  |  |  |  |  |
| **MDA-MB-468** | EGFR | Amplification |  |  |  |  |  |  |  |  |  |
| **SW626** | *Control* |  |  |  |  |  |  |  |  |  |  |
| **Colour legend** | | | | | | | | | | | |
| Mutation did not pass the Q30 filter of the quality control | | | | | | | | | | | |
| Mutation is not described as pathogenic in literature | | | | | | | | | | | |
| Cell line is used as a negative control (HER wild-type) | | | | | | | | | | | |
| The role of the mutation in cancer is debatable. The mutation is frequently observed in the healthy population, as indicated by the dbSNP database (rs1136201). | | | | | | | | | | | |
|  | | | | | | | | | | | |

**Supplementary Figure 1:** Sensitivity of HER-altered cell lines compared to HER wild-type cell lines. Boxplots of HER-altered versus HER-wildtype cell line response to (A) Neratinib, (B) Lapatinib, and (C) Tucatinib. Significance was determined by Mann-Whitney U-test. ** *p*-value < 0.01. *** *p*-value < 0.001. Waterfall plots of ^10^log IC_50_ values relative to the geometric mean for (D) Neratinib, (E) Lapatinib, and (F) Tucatinib. Sensitivity decreases as the waterfall plot descends. Colors indicate alterations in *EGFR* (yellow), *HER2* (red), and *HER3* (blue). Geometrically averaged IC_50_ values are 1.0 µM for Neratinib, 7.1 µM for Lapatinib, and 14.7 µM for Tucatinib.


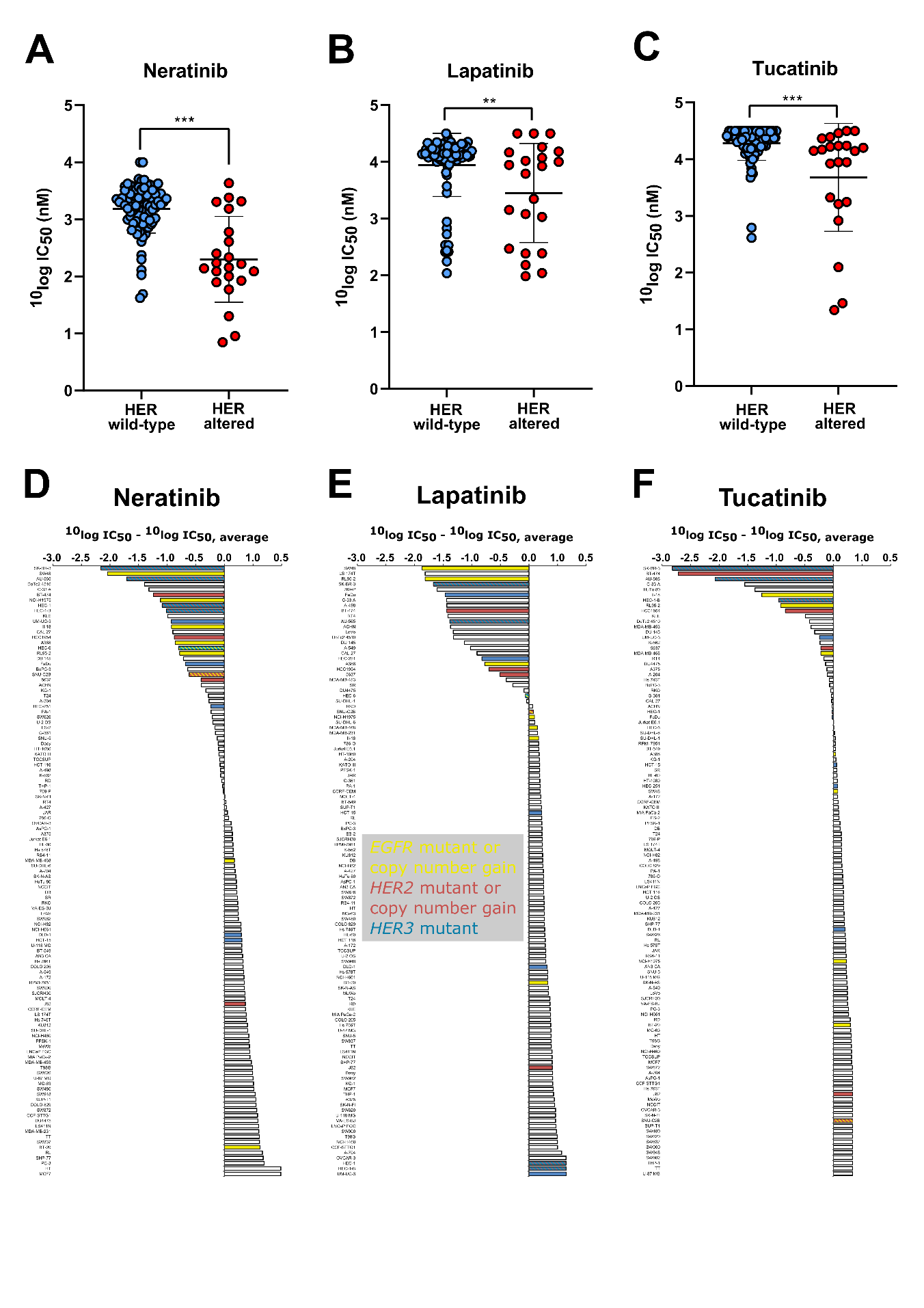


**Supplementary table S4:** Gene set enrichment analysis on the 18,900 correlations with neratinib, lapatinib, and tucatinib responses.

**Ethical Approval and Consent to Participate**

115 cancer cell lines was utilised in this study. All cancer cell lines were purchased from the American Type Culture Collection (ATCC) (Manassas, VA), except II-18, which was acquired from RIKEN BioResource Research Center (Tsukuba, Ibaraki, Japan), and HEC-1, HEC-6 and HEC-251, which were purchased from Japanese Collection of Research Bioresources Cell Bank (JCRB) (Ibaraki city, Osaka, Japan).
